# Diagnostic and prognostic role of peritoneal CA 125 in peritoneal dialysis patients presenting with acute peritonitis

**DOI:** 10.1186/1471-2369-15-149

**Published:** 2014-09-12

**Authors:** Kwanpeemai Panorchan, Andrew Davenport

**Affiliations:** UCL Centre for Nephrology, Royal Free Hospital, University College London Medical School, Rowland Hill Street, NW3 2PF London, UK

**Keywords:** Peritonitis, CA125, Gram + ve, Gram-ve, Peritoneal dialysis

## Abstract

**Background:**

Cancer antigen 125 (CA125) is made by peritoneal mesothelial cells and can be measured in spent dialysate effluent from peritoneal dialysis (PD) patients. It has been suggested that CA125 is a marker of peritoneal mesothelial cell mass and turnover. As PD CA125 increases during peritoneal inflammation, we wished to determine whether measuring PD CA125 during peritonitis provided additional information in determining outcome of peritonitis.

**Methods:**

We prospectively measured peritoneal CA125 in 127 adult PD patients presenting with 187 acute episodes of PD peritonitis, measuring peritoneal CA125 from a sample of dialysate effluent obtained from a 4 hour 2 litre 13.6 g/l dextrose peritoneal dwell.

**Results:**

Mean patient age 60.8 ± 17.1 years, 62.6% male, 33.7% diabetic and the median PD vintage was 22 (11-48) months. 127 patients (66.8%) presented with their first episode of peritonitis, 20% their second episode, 13.2% third or greater. Gram positive bacteria accounted for 64.7% of all peritonitis episodes and Gram negative bacteria 21.1%. Treatment was successful for 151 episodes of PD peritonitis (81.1%). The median PD effluent total WBC was 1240 (430-3660) /ml and serum CRP 67 (20-144) mg/l, with a PD CA125 of 38 (20.3-72.3) IU/l on presentation. There were positive correlations between PD effluent CA125 concentrations and total WBC on presentation (r = 0.41, p = <0.001) and dialysis vintage (r = -0.43, p < 0.001) but not with patient age, diabetic status, or serum CRP.

There was no difference in PD effluent CA125 concentrations between Gram positive, and Gram negative peritonitis or between those episodes which responded to treatment, median 38 IU/ml (21-69) vs those with treatment failures 38 IU/ml (15-94).

**Conclusion:**

We did not find any additional diagnostic or prognostic benefit for measuring effluent CA125 in PD patients presenting with acute peritonitis compared to standard investigations, including peritoneal WBC and serum CRP. As such our study would not support the routine measurement of peritoneal CA125 during episodes of peritonitis.

## Background

Cancer antigen 125 (CA125) is a glycoprotein that has been widely used to screen for ovarian cancer. Subsequently, human mesothelial cells were also demonstrated to secrete CA125, and CA125 has been proposed to reflect mesothelial cell mass in stable peritoneal dialysis (PD) patients [1], and this led to the growing popularity of measuring CA125 as a surrogate biomarker of mesothelial cell mass, peritoneal function and peritoneal membrane preservation [2–4]. However it was soon recognised that peritoneal dialysate effluent CA125 concentrations decline with time on PD and also with encapsulating peritoneal sclerosis [4, 5], and yet on the other hand intra-peritoneal inflammation can lead to an increase in peritoneal CA125 [6]. Indeed it was suggested that peritoneal CA125 could be used to monitor the effects of inflammation with the peritoneum, as after increasing concentrations then fell with resolution of the peritonitis episode [6]. PD peritonitis is the most common cause of PD technique failure [7, 8], and although reports have claimed that the initial peritoneal dialysate effluent white cell count can predict which episodes will respond to appropriate treatment [9], other studies have suggested that the day 3 peritoneal dialysate effluent cell count has greater predictive value [10], whereas others have reported no association between illness severity scores, including peritoneal white cell counts and micro-organism were not predictive of subsequent outcomes [11]. As such we decided to assess the possible diagnostic yield and prognostic value of peritoneal dialysate effluent CA125measurements in cases of acute PD peritonitis.

## Methods

We prospectively measured peritoneal CA125 as we had hypothesised that peritoneal dialysate effluent concentrations may reflect the severity of intraperitoneal inflammation and predict modality outcomes [12, 13]. CA125 was measured using a sandwich immunoassay with chemi-luminescence detection (Roche Diagnostics Modular Analytics E170 analyser) in 127 adult PD patients under the care of a university teaching hospital presenting with 187 acute episodes of acute PD peritonitis between 2005 and 2013. PD peritonitis was defined as > 100 WBC/ml with > 50% polymorphonuclear cells recovered from a standard 2 litre 13.6 g/l dextrose peritoneal dialysate instilled for a 4 hour dwell in PD patients presenting with a cloudy PD effluent, and or signs and symptoms of abdominal pain. Corresponding 30-50 ml peritoneal dialysate effluent samples were also taken for standard microscopy and microbacteriology laboratory cultures and in addition peritoneal dialysate effluent was also inoculated into BD Bactec™ Plus Aerobic/F and Plus Anaerobic/F Mediumand Bactec™ automated microbiology growth and detection system (Beckton Dickson and Company, Plymouth, UK). Peritoneal dialysate effluents were also sent for cytospin analysis with cell staining to confirm both acute inflammatory changes and cell types. A sample of the peritoneal dialysate effluent from the 4 hour 13.6 g/l dextrose dwell was taken for peritoneal CA125 measurement. Serum CA125 was measured using the same immunoassay as for peritoneal CA125, and serum C reactive protein (CRP) by the same method as the UK National Amyloid Centre.

### Approvals

This retrospective audit complied with both the local Royal Free Hospital Research and Development office and the UK NHS guidelines for clinical audit and service development, available at http://www.hra.nhs.uk/documents/2013/09/defining-research.pdf, and http://www.gov.uk/government/publications/health-research-ethics-committees-governance-arrangements.

### Statistical analysis

Data is presented as mean ± standard deviation, or median and interquartile range, unless otherwise stated. Statistical analysis used SPSS version 20 (SPSS, Univ Chicago, USA) and Prism version 6.0 (GraphPad, San Diego, USA) employing student’s t test and Man Whitney U test, Spearman’s correlation, and anova using Kruskal Wallis test with post hoc analysis where appropriate. Multiple linear correlation analysis was performed to determine which variables were associated with PD CA125, with nonparametric variables log transformed, then using a step backwards model, initially including all variables with p < 0.1 on simple correlation, and then eliminating variables that were not significant, or had 95% confidence limits crossing the line of unity unless they did not increase the statistical fit of the model. Receiver operator curves were used to analyse the effect of PD CA125 on technique survival. Statistical analysis was taken at p <0.05.

## Results

The mean age of patients presenting with PD peritonitis was 60.8 ± 17.1 years (range 20-92 years), 62.6% male, 33.7% diabetic and median PD vintage was 22 (11-48) months. 127 patients (66.8%) presented with their first episode of peritonitis, 20% their second episode, 6.8% their third, 3.2% their fourth, and 3.2% with their fifth or greater. Gram positive bacteria accounted for 64.7% (123) of all peritonitis episodes, Gram negative bacteria 21.1% (40), culture negative peritonitis (CNS) 9.5%, fungal peritonitis 2.6% and mixed organism peritonitis in 2.1%. Treatment was successful for 151 episodes of PD peritonitis (81.1%), with treatment failures (catheter removal, recurrence, relapse, or death) in 36 patients (18.9%). Five patients (2.6%) died.

The median PD effluent total WBC was 1240 (430-3660)/ml on presentation and 37 (11.3-91)/ml by day 3. The serum CRP on presentation was 67 (20-144) mg/l, and 58 (22-148) mg/l on day 3, with a maximum CRP of 131 (46-236) mg/l. The median peritoneal dialysate CA125 on presentation was 38 (20.3-72.3) U/l with a serum CA125 15 (11-26) U/l, giving a ratio or peritoneal/serum CA125 of 1.85 (1.1-3.2).

**Figure 1:**
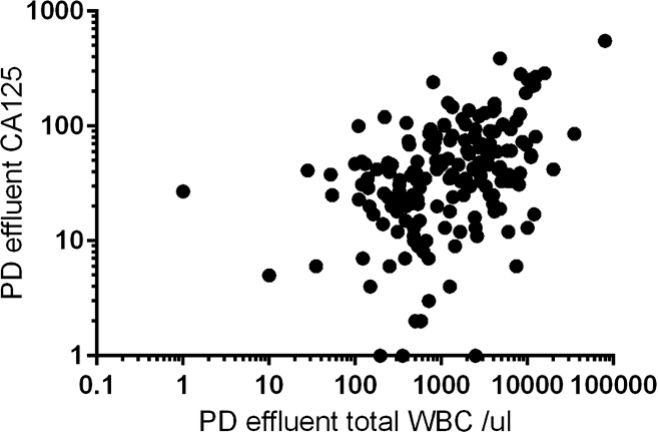
**Correlation between log total peritoneal dialysate effluent white cell count (cells/ul) and CA-125.** Both log values to base 10, Spearman correlation r = 0.41 p <0.001.

There were positive correlations between peritoneal dialysate effluent CA125 concentrations and total white cell count on presentation (r = 0.41, p = <0.001) (Figure 1), and also with the day 3 peritoneal dialysate total WBC (r = 0.37, p < 0.001). Peritoneal CA125 also had simple correlations to both dialysis vintage (r = -0.43, p < 0.001) and serum CA125 (r = 0.59, p = 0.001) but not with patient age, diabetic status, serum CRP on presentation, day 3 or maximum CRP. On multiple linear correlation peritoneal dialysate effluent CA125 correlated with both dialysis vintage (β coefficient -0.27, 95% confidence limits -0.394 to -0.148, standard error 0.057, t statistic -4.36, p < 0.001) and PD effluent total white blood cell count (β coefficient 0.221, 95% confidence limits 0.13 to 0.312, standard error 0.046, t statistic -4.79, p < 0.001), r^2^ for model 0.23, and adjusted r^2^0.22.

**Figure 2:**
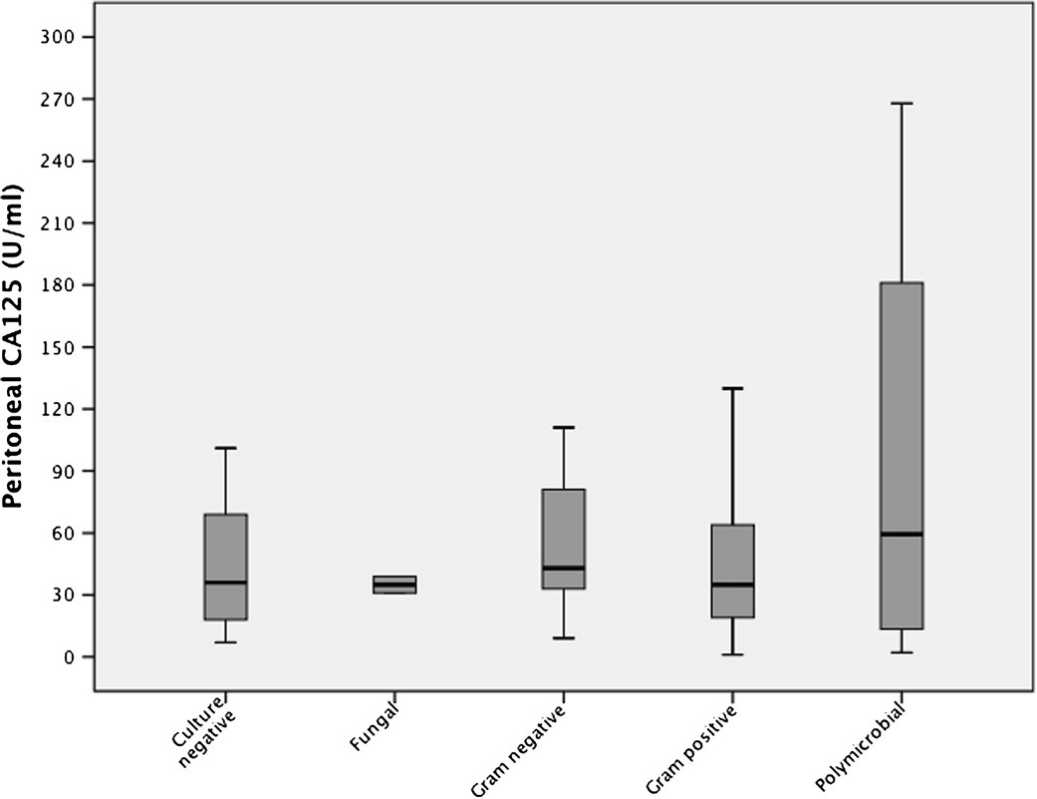
**Box and whisker plot of median, interquartile and 95% confidence limits of peritoneal dialysate CA125 effluents measured on presentation of PD peritonitis according to underlying bacterial infection.** P > 0.05by Kruskal Wallis anova.

**Figure 3:**
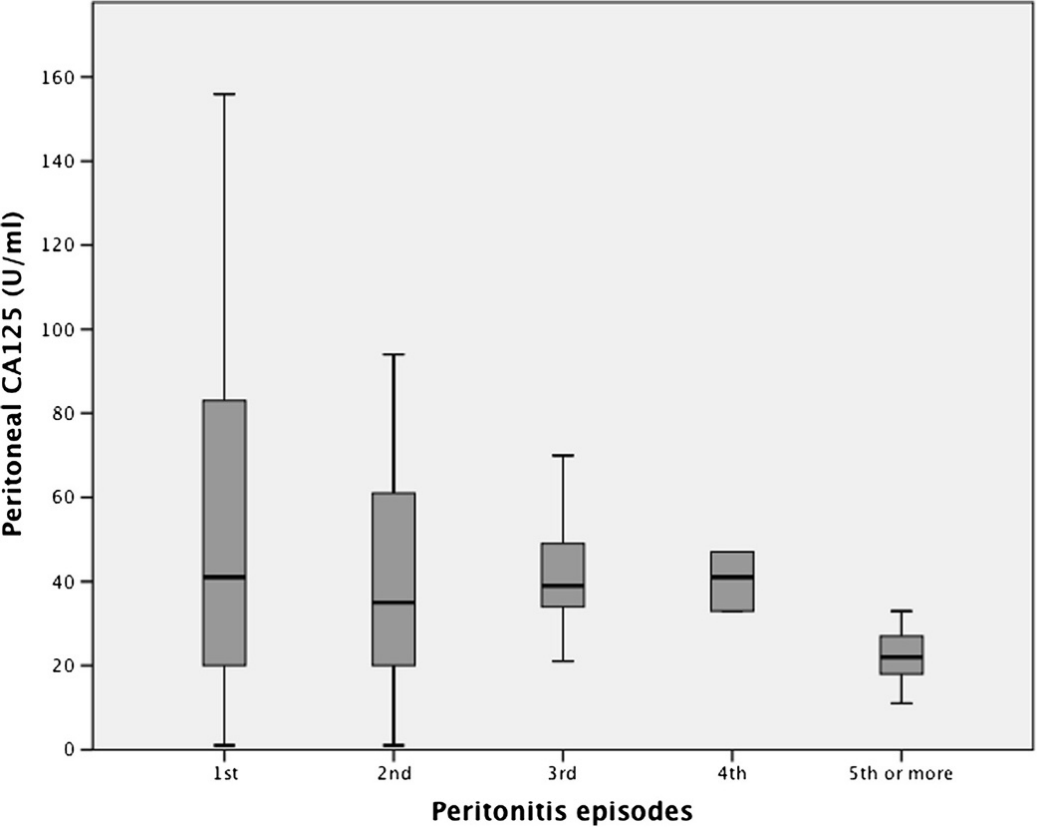
**Box and whisker plot of median, interquartile and 95% confidence limits of peritoneal dialysate CA125 effluents measured on presentation of PD peritonitis according to whether patients experienced 1** ^**st**^ **, 2** ^**nd**^ **, 3** ^**rd**^ **, 4** ^**th**^ **, 5** ^**th**^ **or greater number of peritonitis episode.** P for trend >0.05.

There was no difference in PD effluent CA125 concentrations between Gram positive, Gram negative and culture negative peritonitis (Figure 2). There was a non-significant trend for PD effluent CA125 to decline with increasing number of peritonitis episode (Figure 3). There was no difference in PD effluent CA125 concentrations between those episodes which responded to treatment, median 38 IU/ml (21-69) vs those who suffered treatment failures, 38 IU/ml (15-94) (Table 1). Similarly CA-125 peritoneal dialysis effluent concentrations on presentation did not predict short or longer term PD survival (p = 0.847, r = .-0.014, and p = 0.835, r = -0.015 respectively), nor did the ratio of peritoneal to serum CA125 (p = 0.249, r = 0.132 and p = 0.29, r = 0.120 respectively).

**Table 1:** **peritoneal dialysis CA125 concentrations (IU/L) from effluent of 4 hour 13.6 g/dl dextrose peritoneal dialysate exchange and outcomes assessed as whether patients remained on peritoneal dialysis at 3 and 6 months (PD+), died or transferred to hemodialysis (PD-)**

Organisms	3 months PD+	3 months PD-	6 months PD+	6 months PD-
**Gram + ve**	**37 (23-68)**	**30 (9-80)**	**35 (23-64)**	**40 (11-82)**
**Gram -ve**	**54 (22-75)**	**49 (33-123)**	**49 (20-75)**	**52 (33-108)**
**Culture negative**	**25 (16-44)**	**57 (14-100)**	**30 (15-46)**	**20 (14-100)**
**Fungal**	**-**	**35 (18-59)**	**-**	**35 (18-59)**
**Polymicrobial**	**28 (25-31)**	**135 (2-268)**	**31 (31-31)**	**25 (2-268)**

Patients were censored at time of transplantation. Data expressed as median (interquartile range). All fungal infections resulted in catheter removal. No statistical differences.

A receiver operator curve (ROC) analysis performed to determine whether PD effluent CA125 had any determining effect on peritoneal dialysis technique survival. In neither case was PD CA125 found to have any significant impact for either early or late peritoneal dialysis technique survival (p = 0.87, p = 0.71 respectively), and ROC area 0.52, p = 0.707.

## Discussion

Characterisation of human peritoneal mesothelial cells shows that these mesenchymal cells [14] can produce CA125 [15]. In keeping with earlier smaller studies, peritoneal dialysate effluent CA125 was increased when PD patients presented with acute episodes of PD peritonitis [6]. In our study there was a correlation between effluent CA125 and total white cell counts, suggesting that the increased CA125 may reflect local intra-peritoneal mesothelial cell inflammation and increase cell turnover [6], but not systemic inflammation as there was no correlation with serum CRP. Although there were statistically significant correlations between peritoneal dialysate effluent CA125 and WBC, the r2 values were somewhat low, as such only 13.7-16.8% of the variance in peritoneal dialysate effluent CA125 concentrations could be explained by WBC, so conversely 83.2-86.3% of the explanation lies elsewhere than effluent WBC. Other reports observed a reduction in PD effluent CA125 with dialysis vintage in stable PD patients [4], we also found that PD effluent CA125 was lower in patients with longer peritoneal dialysis vintage during PD peritonitis. Although cell culture studies have reported that donor age of human mesothelial cells affects CA25 release [15], we found not effect with patient age. We did however observe a relationship between serum and peritoneal CA125 concentrations. As such some of the CA125 measured in the peritoneal dialysate effluent may have come from the systemic circulation.

We did not find any differences in peritoneal CA125 effluent concentrations and the cause of peritonitis, with similar results obtained from patients with Gram + ve, Gram-ve, fungal and polymicrobial infections. Similarly there were no differences in peritoneal effluent CA125 from patients who responded successfully to intra-peritoneal antibiotics and those who suffered treatment failures. Although some studies have reported increased peritoneal dialysate effluent WBC on presentation associated with treatment failure [9], this has not been a universal finding [10, 11]. Indeed studies using a disease severity score, including microbacteriology results and patient demographics could not show an association with outcome [11]. As such our finding that peritoneal dialysate effluent CA125 was not affected by the cause of peritonitis and did not predict clinical outcome is not untoward.

Whereas studies have suggested that repeated episodes of PD peritonitis lead to an increased intra-peritoneal inflammatory response and increased risk of catheter removal [16, 17], we did not observe any increase in peritoneal dialysate effluent CA125. Indeed, if anything effluent CA125 concentrations tended to decline with increasing number of infections, probably due to the confounding effect of increasing dialysis vintage.

Our study does not appear to confirm the results of the smaller earlier studies that effluent CA125 concentrations may have had a prognostic role in PD peritonitis [6], as effluent peritoneal CA125 concentrations following a 4 hour dwell were not significantly different according to clinical outcomes. Peritoneal CA125 as a prognostic indicator may lack sensitivity and specificity, as increased peritoneal concentrations could be increased due to reversible mesothelial cell injury, but also secondary to mesothelial cell death and shedding [18]. As such peritoneal CA125 cannot differentiate between reversible and irreversible mesothelial cell damage and as such have limited association with clinical outcomes. In addition systemic CA125 could add to local peritoneal CA125 during active peritoneal inflammation. Studies which have serial measured PD effluent CA125 have described a later second peak in PD CA125 concentrations between 4 and 6 days post peritonitis presentation suggesting peritoneal repair with mesothelial cell regeneration [6].

## Conclusions

Despite some 30 years since the original studies reporting peritoneal CA125 as a biomarker of peritoneal membrane integrity in peritoneal dialysis patients, a solid understanding of the biologic significance of peritoneal CA125 remains to be elucidated, and many questions remain unanswered [19]. We did not find any additional diagnostic or prognostic benefit for measuring effluent CA125 in PD patients presenting with acute peritonitis compared to standard investigations, including peritoneal white blood cell count and serum CRP. As such we would not recommend the routine measurement of peritoneal CA125 during episodes of peritonitis.

## Abbreviations

CA125: Cancer antigen 125
CRP: C reactive protein
NHS: National Health Service
PD: Peritoneal dialysis
WBC: White blood cells
UK: United Kingdom.
